# Strengthening ethics in clinical research

**Published:** 2011-03

**Authors:** Arvind Pandey, Abha Aggarwal, S.D. Seth, Mohua Maulik, Atul Juneja

**Affiliations:** Clinical Trials Registry, National Institute of Medical Statistics ICMR, New Delhi 110 029, India

Sir,

Of late, a number of news articles have appeared in leading newspapers of the country with regard to irregularities in the conduct of clinical trials in India. Notable among these are dubious consent taking procedures^1^and allegations of drug trials on the poor^2^. According to a report published in the Bulletin of the World Health Organization, fewer than 40 Ethics Committees in India are properly constituted and functioning. It is also a matter of concern that there is no legal requirement for investigators or members of the Ethics Committees to declare a conflict of interest^3^. These issues continue to highlight the importance of transparency, accountability and accessibility of clinical trials and their results. The Clinical Trials Registry – India (CTRI) (*www.ctri.nic.in*), an online system for registration of clinical trials, not only establishes an unbiased, scientific public record of clinical trials but has also ushered in an era of transparency accountability and accessibility of clinical trials being conducted in the country^4^–^6^.

The experiences gained through the process of clinical trial registration on CTRI have highlighted the issues which could be addressed for strengthening or augmenting ethics in clinical research.

First and foremost among these is the need for ensuring that guidelines for constitution and functioning of Ethics Committee (EC) are implemented. As part of the routine verification and validation process of trials submitted to the CTRI, trial registrants are required to submit the relevant Ethics Committee approval documents. Review of these documents reveals the extent of improper functioning of several ECs, *e.g.*, it has been noticed that in some of the cases, the trial’s PI or contact person for scientific and public query, is also the member secretary of the EC and signing authority of the EC approval possibly because of the ignorance of the composition of EC.

On the other hand, many academic institutions might not have a proper Ethics Committee for reviewing and approving clinical trials that are being conducted as a part of the postgraduate medical courses not realizing the fact that principles of ethics remain the same for the global trial or for just a dissertation for academic purpose.

Another case in need of attention is the functioning of Independent Ethics Committees (IEC). The mechanism and credibility of a central Independent Ethics Committee granting ethical approval for clearing trials to be conducted at far off distant cities must be re-visited as the EC may not be conversant with the field situation.

The conflict of interest is an important issue which needs attention. It may be made mandatory to declare the conflict of interest- needless to mention an issue difficult to evaluate.

It has been noted that many PIs are involved in multiple trials, *e.g.*, a particular PI is involved in as many as 25 clinical trials. There is a need to be concerned about the workload on PI- the number of trials an investigator is able to handle to do justice with the research.

There is justifiable concern among Indian regulatory authorities that “global multicentre trials” are just a front and actually a majority of patients, are intended to be recruited from India, raising ethical concerns. Global trials registered in the CTRI, are required to declare the number of patients proposed to be recruited from India. Based on the information through CTRI, there have been certain instances where there has been a bias in allocation of recruitment of subjects for the Indian arm in global clinical trials. In these instances it was observed that more than 80 per cent of the recruitment took place from India as against the planned equal allocation.

## References

[1] Gupta S Bhopal gas victims used as guinea pigs: NGO. cms.

[2] Naveen P 100 docs under scanner for drug trials on poor. Hindustan Times 2010; July 25.

[3] (2008). Clinical trials in India: ethical concerns. *Bull World Health Organ*.

[4] Pandey A, Aggarwal AR, Seth SD, Maulik M (2010). Clinical Trials Registry - India: Raising the veil. *Natl Med J India*.

[5] Pandey A, Aggarwal AR, Maulik M, Seth SD (2009). Clinical trial registration gains momentum in India. *Indian J Med Res*.

[6] Pandey A, Aggarwal AR, Seth SD, Maulik M, Bano R, Juneja A (2008). Clinical Trials Registry - India: Redefining the conduct of clinical trials. *Indian J Cancer*.

